# Varicella Zoster meningitis as a mimicker of pseudotumor cerebri in an immunocompetent patient: A case report and literature review

**DOI:** 10.5339/qmj.2022.21

**Published:** 2022-06-04

**Authors:** Yousra Ali, Mhd Baraa Habib, Abeer Safan, Bisher Sawaf, Abdelnaser Elzouki

**Affiliations:** ^1^Internal Medicine Department, Hamad Medical Corporation, Doha, Qatar E-mail: mhabib2@hamad.qa; ^2^Neurology Department, Hamad Medical Corporation, Doha, Qatar

**Keywords:** Herpes Zoster; Meningitis; Viral; Headache; Intracranial hypertension

## Abstract

Varicella-zoster (VZ) meningitis is uncommon in patients with immunocompetence and usually presents with typical rash and fever. However, VZ meningitis can rarely present with symptoms of intracranial hypertension without the classic manifestations. Herein, we describe a 17-year-old female teen who presented with intractable headache and vomiting and diagnosed with VZ meningitis. Her symptoms remarkably improved after a lumbar puncture and acyclovir therapy.

## Introduction

Varicella-zoster virus (VZV) remains latent in the cranial ganglia and dorsal root ganglia after the resolution of the primary infection – varicella (chickenpox); virus reactivation in tissues causes zoster (shingles) disease and manifests commonly with fever and rash.^1^ The central nervous system (CNS) complications of VZV include aseptic meningitis, encephalitis, cerebral infarcts secondary to concomitant granulomatous vasculitis, myelitis, and multiple cranial neuropathies.^2^ Aseptic meningitis is a rare entity. CNS manifestations of VZV infection in patients with immunocompetence are usually detected in cerebrospinal fluid (CSF) samples by polymerase chain reaction (PCR).^3^ Contrary to its common clinical presentation in association with a vesicular rash (exanthem), we herein present an atypical scenario of VZV meningitis manifesting with intracranial hypertension in a young female patient with immunocompetence.

## Case report

A 17-year-old female patient, with unknown chronic illnesses, presented three times within 10 days with a history of a progressive, throbbing holo-cranial headache that progressed rapidly from mild to severe with nausea and repetitive vomiting. It was persistent and had minimal response to simple analgesia (acetaminophen and ibuprofen). She had no fever, rash, visual changes, hearing problems, or any focal neurological symptoms. She had no previous medical or surgical history and had never received regular medications. Family history was noncontributory. She had up-to-date immunizations; however, she was unsure if she received a vaccination against VZV. She was also unsure about prior history of chickenpox infection. On admission, she was in pain of 10/10 in severity, afebrile with normal blood pressure of 117/78 mmHg, and heart rate of 89 beat/minute. Physical examination revealed normal neurological findings and negative meningeal signs (Kernig's/Brudzinski's), whereas fundoscopy was not performed (was missed). Laboratory test results were normal (Table 1). Magnetic resonance imaging and magnetic resonance venography of the head did not detect signs of venous sinus stenosis or radiological features of intracranial hypertension.

Lumbar puncture revealed clear CSF with elevated opening pressure (29 cm-water). The procedure was performed in the lateral recumbent position, and the needle was inserted between L3 and L4. Then, the manometer tube was connected. Subsequently, the pressure was measured after the nadir of fluid meniscus was oscillating at the level of 29 cm-water. Approximately 45 mL of CSF was collected. The patient mentioned a remarkable improvement in her headache after the procedure. CSF analysis showed lymphocytic predominant pleocytosis (Table 1). The diagnosis of VZ meningitis was established based on the positive PCR result of the CSF. Based on expert opinion, the patient received a 10-day course of acyclovir 750 mg 8-hourly administered intravenously (IV) with remarkable improvement.

## Discussion

VZV reactivation causes various neurologic manifestations including acute retinal necrosis, herpes zoster ophthalmicus, post-herpetic neuralgia, myelitis, meningoencephalitis, and VZV vasculopathy.^4^ An elevated intracranial pressure secondary to viral infection is a rare presentation of viral meningitis.^5^ It is distinguished primarily from idiopathic intracranial hypertension (pseudotumor cerebri) by characteristic pleocytosis in the CSF sample, which defies the diagnostic modified Dandy's criteria for pseudotumor cerebri.^6^ Subclinical meningeal irritation secondary to VZV reactivation occurs in 40%–50% of cases reported. The increasing incidence is attributed to the development of diagnostic methods and frequent clinical suspicion even in the absence of common cutaneous clinical manifestations.^7^ Some factors are related to VZV reactivation including family history, female sex, physical trauma, and concomitant comorbidities (such as diabetes, cardiovascular disorders, and renal diseases), besides autoimmune and inflammatory conditions such as systemic lupus erythematosus, rheumatoid arthritis, and inflammatory bowel disease.^8^

The immune system plays a significant role; specifically, memory T cells mediate cellular immunity against various microorganisms, which declines with aging and immune suppression causing viral renaissance.^9^ Only a few reports have described VZV meningitis mimicking idiopathic intracranial hypertension in patients with immunocompetence in the absence of cutaneous manifestations (Table 2). Headache is the chief presenting complaint in all cases. The diagnosis of viral meningitis was established by viral PCR from CSF samples with negative cultures. Moreover, pleocytosis in CSF was observed in all samples. In VZV, although PCR analysis of CSF is considered very sensitive in the first 7–10 days after infection with specificity of 95%,^10^ virus-specific antibodies become readily detectable after this period, and PCR analysis may reveal negative results instead.^11^

All cases demonstrated increased CSF opening pressure, i.e., between 29 and 60 cm in water, establishing a diagnosis of intracranial hypertension in association with VZV meningitis.^12–15^ The first case report has demonstrated a false localizing sign of 6th nerve palsy and papilledema in association with intracranial hypertension in comparison with other cases reported in the literature including our case (Table 2). The association of elevated intracranial pressure in cases of bacterial meningitis, CNS tuberculous, and fungal infections is well-described in the literature in comparison with viral meningitis.^12^ Although current literature lacks a summative list of the common viral etiologies leading to increased ICP, human immunodeficiency virus, measles, VZV, and very rarely enterovirus are the commonly described agents in a survey of existing case reports.^13^ However, the mechanism is poorly understood. This is possibly attributed to one of the proposed theories, as suggested by Lo et al., i.e., post-infective or “allergic” response and diffuse brain swelling due to VZV meningitis or as super-imposed phenomena on a baseline subclinical pseudotumor cerebri.^16^ Patients with elevated ICP and a CSF profile of infectious picture, but has no well-known infectious causes, may represent a subgroup of nonspecific viral syndrome, as referred to by Ravid et al.^17^

Acetazolamide is the standard treatment for pseudotumor cerebri, as it decreases CSF production with efficacy up to 75%^18^; however, its use is often limited because of side effects, which include paresthesia, polyuria, and fatigue.^19^ Adjunctive second-line therapies include topiramate, furosemide, and corticosteroids. A symptomatic control is achieved in) IIH mimickers secondary to viral etiology with a treatment regimen of acetazolamide and appropriate antiviral drugs administered intravenously or orally.^20^ A slightly different treatment approach was used by the four reported cases compared with our case.^12–15^ Notably, antiviral therapy given intravenously has resulted in complete recovery, as compared with oral antiviral therapy where partial recovery was demonstrated in one patient using acyclovir intravenously as reported by Kiefer et al. However, in all other reported cases including our case,^12–14^ antiviral therapy administered intravenously resulted in complete recovery, acyclovir was given intravenously in three cases^12,13^ and ganciclovir was administered in one case.^14^

Only two reported cases^12,13^ have received acetazolamide in combination with antiviral therapy; Ibrahim et al. used a dose of 250 mg PO BID. By contrast, antiviral therapy alone was used in the other reported cases, which were comparable to our case, and similar clinical outcomes were obtained (Table 2).

## Conclusions

Despite its rarity, VZV can cause meningitis in patients with immunocompetence as in our case. The typical rash and fever might be absent in some patients who may present with only headache and vomiting. The present case suggests a possible association between VZV and intracranial hypertension; however, more studies are warranted to delineate a better understanding of this pathophysiology. This case highlights the clinical and scientific importance of CSF viral PCR testing in the evaluation of headache syndromes in the case of elevated ICP.

### Declaration of Competing Interest

The authors report no conflict of interest.

### Funding

This research did not receive any specific grant from funding agencies in the public, commercial, or not-for-profit sectors.

### Consent

Informed consent was obtained for the publication of this case.

### Ethics statement

This study was approved by the Medical Research Center of Hamad Medical Corporation.

### Data access statement

Data of this case are available with MBH and YA.

### Acknowledgment

The authors would like to thank the Qatar National Library for funding the open-access fees of this publication.

## Figures and Tables

**Table 1:** Laboratory values during hospitalization.

Detail	Value w/units	Normal range

Blood test results		

WBC	5.6 x10^3^/μL	4.0–10.0

Hemoglobin	12.6 gm/dL	12.0–15.0

Urea	3.0 mmol/L	2.5–7.8

Creatinine	49 μmol/L	54–95

Sodium	135 mmol/L	133–146

Potassium	3.7 mmol/L	3.5–5.3

C-reactive protein	1 mg/L	0–5

Cerebrospinal fluid (CSF) findings		

Color CSF	Colorless	

Appearance CSF	Clear	

WBC CSF	312/μL	0–5

RBC CSF	14/μL	0–2

Neutrophils CSF	1%	0–6

Lymphocyte CSF	94%	40–80

Monocyte CSF	5%	15–45

Varicella zoster virus PCR	Positive	


**Table 2:** Previous case reports of VZV aseptic meningitis presenting with elevated intracranial pressure in comparison with our case.

References	Age (year)	Sex	Presenting symptoms	Meningeal signs	Papilledema	Nerve palsy

1. Our case	17	F	Headache, nausea, vomiting	No	No	No

2. Ibrahim al.^11^	15	F	Headache, nausea, vomiting	No	Yes	Left 6th nerve

3. Kiefer et al.^12^	46	M	Headache, nausea, dizziness, mild photophobia and phonophobia	No	No	No

4. Fazal^13^	44	M	Headache, nausea, vomiting, photophobia and phonophobia	No	No	No

5. Jia et al.^14^	30	M	Headache	No	No	No

WBC, lyphocytic predominance	Protein	Culture	Causative agent	Opening pressure in cm-water	Treatment	Outcomes

312	46 mg/dL	Negative	VZV by PCR	29	IV acyclovir for 10 days	Complete recovery after 10 days

515	0.62 mmol	Negative	VZV by PCR	53	IV acyclovir + oral acetazolamide for 14 days	Complete recovery after 2 weeks

423	132 mg/dL	Negative	VZV by PCR	36	Oral acyclovir for 14 days plus oral acetazolamide up to 18 months	Dramatic improvement, however not complete resolution of headache

864	105 mg/dL	Negative	VZV by PCR	60	IV ganciclovir for 3 days and was transitioned to oral valacyclovir for a total of 10 days	Complete recovery after 10 days

706	144 mg/dL	Negative	VZV by PCR	29	IV acyclovir for 2 days and was transitioned to oral valacyclovir for another 10 days	Headache resolution within 2 days of commending treatment


## References

[1] Gershon AA, Breuer J, Cohen JI (2015). Varicella zoster virus infection. Nature Reviews. Disease Primers.

[2] Platanaki C, Leonidou L, Siagkris D Varicella-zoster virus aseptic meningitis: An atypical presentation in an immunocompetent male patient. Oxford Medical Case Reports.

[3] Chen L, Xu Y, Liu C Clinical features of aseptic meningitis with varicella zoster virus infection diagnosed by next-generation sequencing: Case reports. BMC Infectious Diseases.

[4] Nagel MA, Gilden D Neurological complications of varicella zoster virus reactivation. Current Opinion in Neurology.

[5] Beal JC “Increased intracranial pressure in the setting of Enterovirus and other viral meningitides.”. Neurology Research International.

[6] Friedman DI, Liu G, Digre K Revised diagnostic criteria for the pseudotumor cerebri syndrome in adults and children Author Response. Neurology.

[7] Alvarez JC, Alvarez J Sr, Tinoco J Varicella-zoster virus meningitis and encephalitis: An understated cause of central nervous system infections. Cureus.

[8] Marra F, Parhar K, Huang B, Vadlamudi N Risk factors for herpes zoster infection: A meta-analysis. Open Forum Infectious Diseases.

[9] Laing KJ (2018;). “Immunobiology of varicella-zoster virus infection.”. The Journal of Infectious Diseases September.

[10] Osiro S, Salomon N Varicella-zoster virus (VZV) multifocal vasculopathy in a patient with systemic lupus erythematosus — A diagnostic and treatment dilemma. IDCases.

[11] Skripuletz T, Pars K, Schulte A Varicella zoster virus infections in neurological patients: A clinical study. BMC Infectious Diseases.

[12] Ibrahim W, Elzouki AN, Husain A, Osman L Varicella zoster aseptic meningitis: Report of an atypical case and literature review. American Journal of Case Reports.

[13] Kiefer L, Adam D, Mudugal D, Burnett MS Viral meningitis mimicking benign intracranial hypertension: A report of two cases. Interdisciplinary Neurosurgery.

[14] Raziq FI Varicella zoster virus causing aseptic meningitis without fever or rash in an immunocompetent patient. American Journal of Infectious Diseases.

[15] Jia S, Luong T, Jia S Atypical presentation of aseptic meningitis due to Varicella zoster: A case report. Clinical Practice and Cases in Emergency Medicine.

[16] Lo S, Phillips DIW, Peters JR, Hall M, Hall R Papilloedema and cranial nerve palsies complicating apparent benign aseptic meningitis. Journal of the Royal Society of Medicine.

[17] Ravid S, Shachor-Meyouhas Y, Shahar E, Kra-Oz Z, Kassis I Viral-induced intracranial hypertension mimicking pseudotumor cerebri. Pediatric Neurology.

[18] Johnson LN, Krohel GB, Madsen RW, March GA The role of weight loss and acetazolamide in the treatment of idiopathic intracranial hypertension (pseudotumor cerebri). Ophthalmology.

[19] Schmickl CN, Owens RL, Orr JE, Edwards BA, Malhotra A “Side effects of acetazolamide: A systematic review and meta-analysis assessing overall risk and dose dependence.”. BMJ Open Respiratory Research.

[20] Smith HZ, Paguia R, Horne J, Velagapudi M A case report of human Herpesvirus-6 (HHV-6) meningitis masquerading as idiopathic intracranial hypertension in an immunocompetent patient. Cureus.

